# Reviewer acknowledgement

**DOI:** 10.1186/1749-7922-9-24

**Published:** 2014-03-31

**Authors:** Fausto Catena, Frederick Moore

**Affiliations:** 1 Chief of the Emergency Surgery Department, Parma University Hospital, Parma, Italy; 2 Chief of Acute Care Surgery, Department of Surgery, The University of Florida, Gainesville, Florida

## Abstract

**Contributing reviewers:**

The *World Journal of Emergency Surgery*—which received its first Impact Factor in 2013—is extremely grateful for the time, hard work and support of its highly-qualified peer reviewers. The editors of *World Journal of Emergency Surgery* and BioMed Central would like to show our appreciation by thanking the following people for their assistance reviewing manuscripts for the journal in 2013.

## 

Ferdinando Agresta

Italy

Miklosh Bala

Israel

Chad Ball

Canada

Zsolt Balogh

Australia

Gary Bass

Ireland

Christoph Benckert

Germany

Cino Bendinelli

Australia

Jasneet Bhullar

United States of America

Walter Biffl

United States of America

L.D. Britt

United States of America

Clay Cothren Burlew

United States of America

Osvaldo Chiara

Italy

Ian Civil

New Zealand

Raul Coimbra

United States of America

David Costa Navarro

Spain

Elias Degiannis

South Africa

Marci DeLosSantos

United States of America

Salomone Di Saverio

Italy

Hasan Ekim

Turkey

Alex Escalona

Chile

Paula Ferrada

United States of America

Ralf Herbert Gahr

Germany

Sanjay Gupta

India

Chien-Sheng Huang

Taiwan

Rao Ivatury

United States of America

Gregory Jurkovich

United States of America

Ignatius Kakande

Rwanda

J Kashuk

United States of America

Kenji Kawamukai

Italy

Cuneyt Kayaalp

Turkey

Michael Kelly

Australia

Boris Kirshtein

Israel

Yoram Kluger

Israel

M. Margaret Knudson

United States of America

Sungsoo Lee

South Korea

Luke Leenen

Netherlands

I. Michael Leitman

United States of America

Ari Leppaniemi

Finland

Ronald Maier

United States of America

Mark Malangoni

United States of America

Gyanendra Malla

Nepal

Haridimos Markogiannakis

Greece

Niels Martin

United States of America

Hisashi Matsumoto

Japan

Andrea Mingoli

Italy

Umberto Morelli

France

Sheikh Muzamil

India

Noel Naidoo

South Africa

Marcus Ong

Singapore

Lars Ivo Partecke

Germany

Abhijeet Patil

United Kingdom

Michael Ramdass

Trinidad and Tobago

Sandeep Sainathan

United States of America

Boris Sakakushev

Bulgaria

Thomas Scalea

United States of America

Vishalkumar Shelat

Singapore

Aleksandar Simic

Serbia

Zoe Smith

United Kingdom

Philip Stahel

United States of America

Angela Sultana

Malta

Wieslaw Tarnowski

Poland

Korhan Taviloglu

Turkey

Yoshihito Ujike

Japan

Selman Uranues

Austria

Gabrielle van Ramshorst

Netherlands

Ettore Vulcano

Italy

Imtiaz Wani

India

Dieter Weber

Australia

